# Results of a protocol to limit blood utilization vary based on the cardiac procedure performed

**DOI:** 10.1186/1749-8090-10-S1-A106

**Published:** 2015-12-16

**Authors:** JB Grau, CE Kuschner, F Giovanni, E Tormey, S Wilson, B Mariano, A Zapolanski, RE Shaw

**Affiliations:** 1 Valley Heart and Vascular Institute, Department of Cardiac Surgery, Ridgewood, NJ, USA; 2 University of Pennsylvania, Department of Surgery, Philadelphia, PA, USA

## Background/Introduction

The use of blood transfusions has been shown to increase the risk of mortality and morbidity in cardiac surgery patients.

## Aims/Objectives

To determine whether the implementation of a hospital protocol designed to reduce unnecessary blood transfusions has consistent impact across different cardiac operations.

## Method

From 1/1/2012 through 12/31/2014, 1189 patients underwent isolated coronary artery bypass (IsoCAB, N = 252), isolated valve (IsoValve, N = 190), CAB+Valve (n = 114), CAB+Other (N = 304), Valve+Other (N = 178), or other (151) surgeries. Overall 802 (67.5%) were male with a mean age of 70 ± 12 years. A protocol focusing on reducing blood use was initiated on 11/1/2013. We divided the cohort into two eras, pre-protocol (PreP, n = 736) and post-protocol (PostP, n = 453), to assess the impact of the protocol on transfusion rates in each surgical category.

## Results

There was no significant difference in the type of cardiac surgery, baseline platelet count, Society of Thoracic Surgeons mortality or reoperation risk, or Off-Pump approach in CABG patients between the PreP and PostP groups. Fewer patients in the PostP group received any blood products (45.5% vs. 38.2%; p = 0.008) and fewer PostP patients received 1 unit or 2 or more units of blood (p = 0.045). However, when assessing blood utilization by procedure, only PostP patients undergoing IsoCAB or CAB+Other had significantly reduced utilization of any blood product (37.7% vs. 14.9%; p = 0.001 and 33.9% vs. 24.1%; p = 0.048). When PostP IsoCAB and CAB+Valve patients were transfused, they received a significantly greater number of units of blood (2.51 vs. 3.73; p = 0.082 and 4.77 vs. 9.39; p = 0.017). There was no difference in mortality between the PreP and PostP groups (2.4% vs. 3.5%; p = 0.180).

## Discussion/Conclusion

Cardiac operations involving coronary artery bypass grafting may be most readily targeted to limit blood transfusions. Future protocols should recognize that different cardiac operations hold unique transfusion requirements that will respond differently to the implementation of a protocol driven approach to blood transfusion.

